# Evaluation of HIV testing recommendations in specialty guidelines for the management of HIV indicator conditions

**DOI:** 10.1111/hiv.12430

**Published:** 2016-08-18

**Authors:** E Lord, AJ Stockdale, R Malek, C Rae, I Sperle, D Raben, A Freedman, D Churchill, J Lundgren, AK Sullivan, the British Association of Sexual Health HIV (BASHH)/British HIV Association (BHIVA) guideline review group for the Optimising Testing and Linkage to Care for HIV across Europe (OptTEST) project by HIV in Europe, J. Kabel, K. Block, V. Delpech, A. Sullivan, R. Lowbury, Y. Yazdanpanah, J. Hows, J. Del Amo, K. Rüütel

**Affiliations:** ^1^ Department of Sexual HealthChurchill HospitalOxford University Hospitals NHS TrustOxfordUK; ^2^ Institute of Infection and Global HealthUniversity of LiverpoolLiverpoolUK; ^3^ Imperial College London NHS HealthcareSt Mary's HospitalLondonUK; ^4^ Chelsea and Westminster Hospital NHS Foundation TrustLondonUK; ^5^ RigshospitaletUniversity of CopenhagenCopenhagenDenmark; ^6^ Cardiff University School of MedicineCardiffUK; ^7^ Royal Sussex County HospitalBrighton and HoveUK

**Keywords:** AIDS‐defining conditions, HIV testing, indicator conditions

## Abstract

**Objectives:**

European guidelines recommend HIV testing for individuals presenting with indicator conditions (ICs) including AIDS‐defining conditions (ADCs). The extent to which non‐HIV specialty guidelines recommend HIV testing in ICs and ADCs is unknown. Our aim was to pilot a methodology in the UK to review specialty guidelines and ascertain if HIV was discussed and testing recommended.

**Methods:**

UK and European HIV testing guidelines were reviewed to produce a list of 25 ADCs and 49 ICs. UK guidelines for these conditions were identified from searches of the websites of specialist societies, the National Institute of Clinical Excellence (NICE) website, the NICE Clinical Knowledge Summaries (CKS) website, the Scottish Intercollegiate Guidance Network (SIGN) website and the British Medical Journal Best Practice database and from Google searches.

**Results:**

We identified guidelines for 12 of 25 ADCs (48%) and 36 of 49 (73%) ICs. In total, 78 guidelines were reviewed (range 0–13 per condition). HIV testing was recommended in six of 17 ADC guidelines (35%) and 24 of 61 IC guidelines (39%). At least one guideline recommended HIV testing for six of 25 ADCs (24%) and 16 of 49 ICs (33%). There was no association between recommendation to test and publication year (*P* = 0.62).

**Conclusions:**

The majority of guidelines for ICs do not recommend testing. Clinicians managing ICs may be unaware of recommendations produced by HIV societies or the prevalence of undiagnosed HIV infection among these patients. We are piloting methods to engage with guideline development groups to ensure that patients diagnosed with ICs/ADCs are tested for HIV. We then plan to apply our methodology in other European settings as part of the Optimising Testing and Linkage to Care for HIV across Europe (OptTEST) project.

## Introduction

Despite extensive efforts to promote HIV testing, late diagnosis (CD4 count at diagnosis < 350 cells/μL) 1 continues to be reported in almost half of all newly diagnosed cases in Europe, and 27% of patients diagnosed with HIV infection present with advanced HIV disease (CD4 count < 200 cells/μL) 2. Rates of late diagnosis do not appear to be improving. A meta‐regression of temporal trends in studies reporting CD4 count at diagnosis in Europe and North America showed no significant increase over a 20‐year period between 1992 and 2011 3. Data from European HIV‐infected cohorts have demonstrated no change in median CD4 count at presentation among 30 454 patients from 34 countries between 2010 and 2013 [adjusted change per year 1.2 cells/μL; 95% confidence interval (CI) −0.8 to 3.3 cells/μL] 4.

The importance of early diagnosis is clear: almost all HIV‐associated mortality is attributable to late diagnosis, with a 1‐year relative risk of mortality between 6.6 and 13 in the first year for late diagnoses, depending on European region 1. Diagnosis at a CD4 count of 100–199 or < 100 cells/μL was associated with a mean of 17.8 and 20.9 years of life lost, respectively, in the UK collaborative HIV‐infected cohort, compared with those diagnosed at > 350 cells/μL 5. Initiation of antiretroviral therapy (ART) at a CD4 count > 500 cells/μL is associated with a reduced risk of malignancy, cardiovascular disease and infection 6. Late initiation of ART is associated with poorer treatment responses, as well as persistence of metabolic and inflammatory abnormalities even after years of treatment 7, 8. Additionally, earlier diagnosis presents an opportunity to prevent onward transmission.

The HIV Indicator Diseases Across Europe Study (HIDES 1) recruited patients from 200 health care centres in Europe and offered HIV testing to patients presenting with one of eight indicator conditions (ICs) in order to ascertain the prevalence of undiagnosed HIV infection. Across all eight ICs, the prevalence of HIV infection was 1.8% (95% CI 1.4−2.3%) and ranged from 0.29 to 4.1%; all eight conditions were associated with a prevalence exceeding 0.1% 9. In a subsequent study, HIDES 2, HIV prevalence exceeded a predetermined cost‐effectiveness threshold of 0.1% among patients presenting with 10 of 14 indicator conditions 10. Despite this accumulating evidence, a gap persists between European and national guidelines for testing and implementation, resulting in missed opportunities for diagnosis 11.

European guidelines recommend HIV testing for individuals presenting with AIDS‐defining conditions (ADCs) and ICs (defined as those associated with an undiagnosed HIV prevalence of > 0.1%, or conditions where failure to identify HIV infection may have both health and treatment implications 12). Guidelines from HIV societies inform those working in the HIV community, yet it is other medical specialties that see the majority of patients presenting with ADCs and ICs. It is therefore important to ensure that non‐HIV specialist guidelines for the management of these conditions appropriately recommend HIV testing. Currently, the extent to which such recommendations occur is unknown.

Our aim was to determine the proportion of UK guidelines for ADCs and ICs that appropriately recommend HIV testing and to develop methodology that could be applied to other European countries as part of the Optimising Testing and Linkage to Care for HIV across Europe (OptTEST) project (www.OptTEST.eu). Ultimately, the aim is to identify opportunities to facilitate inclusion of HIV testing recommendations in future guidelines.

## Methods

European (2012) 12 and UK HIV testing guidelines (2008) 13 were reviewed to develop the list of 25 ADCs and 49 ICs 12, 13 (Table S1). We conducted a literature search for relevant UK specialty guidelines for each ADC and IC, including relevant specialty society, association or college websites, the National Institute of Clinical Excellence (NICE) website, the NICE Clinical Knowledge Summaries (CKS) (which provide guidelines for primary care physicians) website, the Scottish Intercollegiate Guidance Network (SIGN) website, the British Medical Journal Best Practice database and Google. HIV‐specific guidelines and those published by the British Association of Sexual Health and HIV (BASHH) were excluded. For example search strategies, please see the Appendix S1.

Each guideline was reviewed and classified into one of three categories: (1) HIV was not mentioned in the guideline; (2) the association with HIV was mentioned but testing was not recommended; (3) HIV was mentioned and testing recommended.

The associations of recommendation to test with categorical variables (source of guideline and type of condition) and with year of publication were tested using Fisher's exact test and Mantel−Haenszel linear‐by‐linear chi square tests, respectively. Data from guidelines were tabulated in a Microsoft Excel database (Microsoft, Redmond, WA, USA). Statistical analyses were conducted using spss version 22 (IBM, Armonk, NY, USA).

## Results

A total of 78 relevant guidelines were identified (range 1–13 per condition); 17 for ADCs (range 0–4) and 61 for ICs (range 0–13) (Table 1). Guidelines were identified for 12 of 25 ADCs (48%) and 36 of 49 ICs (73%) (Table S1).

**Table 1:** Recommendation for HIV testing and reporting of association with HIV, stratified by type of guideline

	Number of guidelines identified (% of total)		Association with HIV reported *n* (%)		HIV testing recommended *n* (%)		*P*‐value for between‐group difference in HIV testing recommendation
All guidelines	78	(100)	41	(53)	30	(38)	
AIDS‐defining conditions	17	(21)	9	(53)	6	(35)	1.0
Indicator conditions	61	(78)	32	(52)	24	(39)	
Source of guideline							
NICE	12	(15)	7	(58)	3	(25)	0.021
NICE Clinical Knowledge Summaries	29	(37)	18	(62)	11	(38)	
SIGN	8	(10)	0	(0)	0	(0)	
Specialty society guidelines	29	(37)	16	(55)	16	(55)	
Guidelines for eight key indicator conditions^a^							
Total	34	(100)	27	(79)	20	(59)	0.002^b^
Sexually transmitted infections	13	(38)	13	(100)	7	(54)	0.20
Malignancy or lymphoma	3	(9)	3	(100)	3	(100)	
Cervical or anal cancer/dysplasia	5	(15)	1	(20)	1	(20)	
Herpes zoster	1	(3)	0	(0)	0	(0)	
Hepatitis B or C virus (acute or chronic)	6	(18)	4	(67)	4	(67)	
Mononucleosis‐like illness	2	(6)	2	(100)	1	(50)	
Unexplained leucocytopaenia, thrombocytopaenia (>4 weeks)	3	(9)	3	(100)	3	(100)	
Seborrhoeic dermatitis/exanthema	1	(3)	1	(100)	1	(100)	

NICE, National Institute of Clinical Excellence; SIGN, Scottish Intercollegiate Guidance Network.

a The eight key indicator conditions were tested as part of the HIDES 1 study and were associated with a prevalence of undiagnosed HIV infection of >0.1%.

b The *P*‐value refers to the comparison between guidelines for the eight key indicator conditions and remaining guidelines.

**Figure 1:**
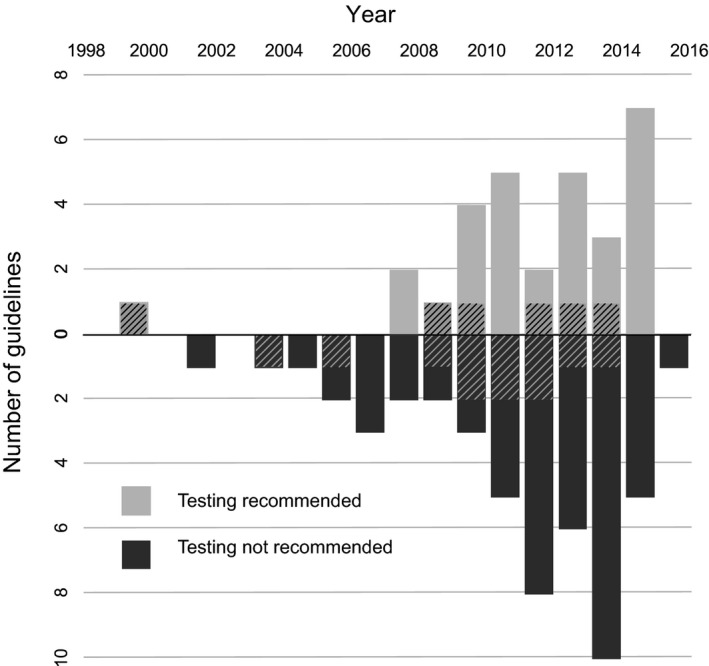
Recommendation for HIV testing in AIDS‐defining conditions (ADCs) and indicator conditions (ICs), stratified by year of guideline publication: no association was observed between publication year and recommendation to test (*P* = 0.620). ADCs are indicated by cross‐hatched boxes.

Association with HIV was discussed in nine of 17 (53%) ADC guidelines and 32 of 61 IC guidelines (52%), whereas HIV testing was appropriately recommended in six of 17 ADC guidelines (35%) and 24 of 61 IC guidelines (39%) (Table 1). At least one guideline recommended HIV testing for six of 25 ADCs (24%) and 16 of 49 ICs (33%). National guidelines from NICE, including clinical knowledge summaries, or from SIGN were less likely to recommend HIV testing than specialist society guidelines (29 vs. 55%, respectively; *P* = 0.02). Guidelines for the eight key ICs identified in the HIDES 1 study were significantly more likely to recommend HIV testing than those for remaining ICs (59 vs. 23%, respectively; *P* = 0.002). No association was observed between year of publication and recommendation to test (*P* = 0.620); see Figure 1.

## Discussion

Only 38% of the guidelines for ADCs and ICs that we identified from UK guidelines recommended HIV testing. While over half of guidelines (53%) acknowledged an association between HIV and the condition, over a quarter of these did not go on to recommend HIV testing. We further identified that the national guideline development bodies NICE and SIGN were significantly less likely to recommend testing compared with specialist society guidelines. The underlying cause of this is unclear. It is important that HIV physicians and National Boards of Health at both European and national levels take the opportunity to engage with guideline development groups to promote testing.

It has been reported that a significant proportion of undiagnosed patients present to health care providers with a clinical episode directly related to HIV 14. Additionally, individuals newly diagnosed with HIV infection report a high level of prior attendance in primary and secondary care where opportunities for earlier diagnosis were missed 15. In a cross‐sectional analysis from a multicentre data set from general practice, 59% of patients diagnosed with HIV infection had exhibited an indicator condition in the 5 years prior to diagnosis compared with 7% among matched controls 16. A lack of awareness surrounding ICs and lack of confidence in offering testing are common barriers among physicians to offering a test 12. Reassuringly, evidence suggests that, despite low offer rates, when an HIV test is suggested, uptake approaches 100% 17. This highlights that the key barrier to HIV testing is that the test is not offered, rather than patients refusing.

Review of guidelines for the eight key indicator conditions included in the HIDES 1 study indicates that a higher proportion did recommend testing. However, several key guidelines failed to do so. Only 54% of sexually transmitted infection (STI) guidelines (all published by NICE) recommend HIV testing (http://cks.nice.org.uk). While it may have been suggested to screen for other STIs, 46% of guidelines failed to explicitly advise that this should include HIV and, while patients attending sexual health clinics are usually routinely screened for HIV, those who present to their GPs may not. Given the increased risk of transmission associated with concomitant STIs, it is particularly important that such guidelines reinforce the need for testing 18. Additionally, patients infected with HIV have a higher incidence of both cervical and anal dysplasia, with increased rates of progression to cancer without treatment 19. However, only 20% of guidelines on cervical and anal dysplasia recommended HIV testing. Mononucleosis‐like illnesses can mimic HIV seroconversion, which occurs in up to 80% of patients who acquire HIV 20. However, only one of the two guidelines identified recommended testing. Patients may present with these symptoms to their GP, and during this highly infectious period a prompt diagnosis is essential. In HIDES 2, 5.3% of patients with suspected mononucleosis tested positive for HIV 10. Patients with ICs may already have late‐stage HIV infection, and failure to test for HIV may have an adverse impact on long‐term prognosis 5. With clear evidence that testing in these situations is cost effective 17, it raises the question as to why testing is not recommended in all relevant evidence‐based guidelines.

Limitations to this project include the lack of established methodology for searching for national guidelines, particularly in comparison to methods for searching biomedical literature; many guidelines are not indexed on biomedical databases. There was also a degree of subjectivity to determination of a testing recommendation; in some cases guidance was ambiguous and this wording should be clarified.

IC‐guided HIV testing is an acceptable, feasible and important part of the strategy to disrupt HIV transmission and promote earlier diagnosis in Europe. Specialists managing ICs may be unaware of national recommendations produced by HIV societies, the prevalence of undiagnosed HIV infection among patient with ICs and the cost of missing opportunities to make an early diagnosis. It is intended that this guideline review process will be extended to other European countries. We are currently developing methods of engaging with guideline development groups to ensure that HIV testing is recommended in future guideline revisions.

## Supporting information


**Table S1**. Number of specialty guidelines identified for each AIDS‐Defining Condition (ADC) and Indicator Condition (IC).

## Appendix

The OptTEST steering committee members are J. Kabel, K. Block, V. Delpech, A. Sullivan, R. Lowbury, Y. Yazdanpanah, J. Hows, J. Del Amo, K. Rüütel, J. Lundgren and D. Raben.
