# Supplementary material for: Evaluation of HIV testing recommendations in specialty guidelines for the management of HIV indicator conditions
Source: HIV Med. 2016 Aug 18;18(4):300–4. doi: 10.1111/hiv.12430 (PMC5347885; doi:10.1111/hiv.12430)
Supplement: Supplementary file 1 — Table S1. Number of specialty guidelines identified for each AIDS‐Defining Condition (ADC) and Indicator Condition (IC). [file HIV-18-300-s001.docx]

**Supplementary table 1: Number of specialty guidelines identified for each AIDS-Defining Condition (ADC) and Indicator Condition (IC)**

| **AIDS DEFINING CONDITIONS (ADCs)** (Range 0-4, overall median 0, interquartile range (IQR 0, 1.5) | | | |
| --- | --- | --- | --- |
| **Neoplastic** | **N** | **Viral Infection** | **N** |
| Cervical cancer | 4 | CMV retinitis | 0 |
| Non-Hodgkin lymphoma | 2 | CMV, other (except liver, spleen, glands) | 0 |
| Kaposi’s sarcoma | 1 | HSV ulcer(s) >1 month/bronchitis/pneumonitis | 0 |
| Primary cerebral lymphoma | 1 | Progressive multifocal leucoencephalopathy | 0 |
| **Bacterial Infection** | | **Parasitic Infection** | |
| MTB, pulmonary or extrapulmonary | 2 | Cerebral toxoplasmosis | 0 |
| MAC or Mycobacterium kansasii, D/EP | 1 | Cryptosporidiosis diarrhoea, >1 month | 2 |
| Mycobacterium, other/unidentified species D/EP | 1 | Isosporiasis, >1 month | 2 |
| Pneumonia, recurrent (≥2 episodes in 12 months) | 2 | Atypical disseminated leismaniasis | 1 |
| Salmonella septicaemia, recurrent | 0 | Reactivation of American trypanosomiasis | 0 |
| **Fungal Infection** | | | |
| Pneumocystis carinii pneumonia | 0 | Histoplasmosis, D/EP | 0 |
| Candidiasis, oesophageal | 1 | Coccidiodomycosis, D/EP | 0 |
| Candidiasis, bronchial/tracheal/lungs | 0 | Penicilliosis, disseminated | 0 |
| Cryptococcosis, extra-pulmonary | 0 |  | |
| **INDICATOR CONDITIONS (ICs)** (Range 0-13, overall median 1 (IQR 0, 2) | | | |
| **Respiratory** | **N** | **Dermatology** | **N** |
| Community acquired pneumonia | 2 | Herpes Zoster | 1 |
| Invasive pneumococcal disease | 1 | Seborrheic dermatitis/ exanthema | 1 |
| Aspergillosis | 0 | Severe or atypical psoriasis | 2 |
| **Neurology** | | **Gastroenterology** | |
| Lymphocytic meningitis | 1 | Hepatitis A | 1 |
| Guillain–Barré syndrome | 0 | Hepatitis B (Acute Or Chronic) | 3 |
| Subcortical dementia | 1 | Hepatitis C (Acute Or Chronic) | 3 |
| Peripheral neuropathy | 1 | Unexplained weight loss | 1 |
| Primary cerebral space occupying lesion | 0 | Unexplained oral candidiasis | 1 |
| Mononeuritis | 1 | Unexplained chronic diarrhoea | 2 |
| Multiple sclerosis-like disease | 1 | Oral hairy leukoplakia | 1 |
| Cerebral abscess | 0 | *Salmonella*, *Shigella* or *Campylobacter* spp. | 1 |
| Transverse myelitis | 0 | **Ear/Nose and Throat (ENT)** | |
| Leucoencephalopathy | 0 | Chronic parotitis | 0 |
| **Opthalmology** | | Lymphoepithelial parotid cysts | 1 |
| Infective retinal diseases | 1 | **Oncology** | |
| Any unexplained retinopathy | 0 | Primary lung cancer | 2 |
| **Haematology** | | Anal cancer/ dysplasia | **1** |
| Malignant lymphoma/Hodgkin's lymphoma | 1 | Cervical dysplasia | 3 |
| Unexplained leukocytopenia/thrombocytopenia lasting >4 weeks | 2 | Vaginal intraepithelial neoplasia | 3 |
| Idiopathic/thrombotic thrombocytopenic purpura | 1 | Seminoma | 2 |
| **Ear/Nose And Throat (ENT)** | | Head and neck cancer | 3 |
| Chronic parotitis | 0 | Castleman's | 0 |
| Lymphoepithelial parotid cysts | 1 | **Renal medicine** | |
| **Other** | | Unexplained chronic renal impairment | 3 |
| Sexually transmitted infections | 13 | Candidaemia | 0 |
| Unexplained fever | 1 | Visceral leishmaniasis | 0 |
| Unexplained lymphadenopathy | 3 | Candidiasis | 0 |
| Mononucleosis-like illness | 2 | Conditions requiring immunosuppressive therapy | 6 |

a Guidelines may relate to >1 ADC/IC. Abbreviations: CMV, Cytomegalovirus; HSV, Herpes simplex virus; MTB= mycobacterium tuberculosis; MAC, Mycobacterium avium complex; D/EP, Disseminated or extrapulmonary;
